# Virologic efficacy of tenofovir, lamivudine and dolutegravir as second-line antiretroviral therapy in adults failing a tenofovir-based first-line regimen: a prospective cohort study

**DOI:** 10.1097/QAD.0000000000002936

**Published:** 2021-07-15

**Authors:** Claire M Keene, Rulan Griesel, Ying Zhao, Zimasa Gcwabe, Kaneez Sayed, Andrew Hill, Tali Cassidy, Olina Ngwenya, Amanda Jackson, Gert Van Zyl, Charlotte Schutz, Rene Goliath, Tracy Flowers, Eric Goemaere, Lubbe Wiesner, Bryony Simmons, Gary Maartens, Graeme Meintjes

**Affiliations:** 1 Médecins Sans Frontières South Africa; 2 Division of Clinical Pharmacology, Department of Medicine, University of Cape Town, Cape Town, South Africa; 3 Wellcome Centre for Infectious Diseases Research in Africa, Institute of Infectious Disease and Molecular Medicine, University of Cape Town, Cape Town, South Africa; 4 University of Cape Town, Cape Town, South Africa; 5 University of Liverpool, Department of Pharmacology, Liverpool, United Kingdom; 6 Division of Public Health Medicine, School of Public Health and Family Medicine, University of Cape Town, Cape Town, South Africa; 7 University of Stellenbosch, Division of Medical Virology, Cape Town, South Africa; 8 Department of Medicine, University of Cape Town, Cape Town, South Africa; 9 Department of Infectious Disease, Imperial College London, London, United Kingdom

**Keywords:** second-line, antiretroviral therapy, dolutegravir, HIV

## Abstract

**Objective:**

Recycling tenofovir and lamivudine/emtricitabine (XTC) with dolutegravir would provide a more tolerable, affordable, and scalable second-line regimen than dolutegravir with an optimized nucleoside reverse transcriptase inhibitor (NRTI) backbone. We evaluated efficacy of tenofovir/lamivudine/dolutegravir (TLD) in patients failing first-line tenofovir/XTC/efavirenz or nevirapine.

**Design:**

Single arm, prospective, interventional study

**Setting:**

Two primary care clinics in Khayelitsha, South Africa

**Participants:**

60 adult patients with two viral loads (VL)>1000 copies/mL

**Intervention:**

Participants were switched to TLD with additional dolutegravir (50mg) for two weeks to overcome efavirenz induction.

**Primary outcome:**

Proportion achieving VL<50 copies/mL at week 24 using the FDA snapshot algorithm.

**Results:**

Baseline median CD4 count was 248 cells/mm^3^, VL 10580 copies/mL and 48/54 (89%) had resistance (Stanford score ≥15) to one or both of tenofovir and XTC. No participants were lost to follow-up. At week 24, 51/60 (85%, 95% CI 73-93%) were virologically suppressed, six had VL 50-100 copies/mL, one VL 100-1000 copies/mL, one no VL in window, and one switched due to tenofovir-related adverse event. No integrase mutations were detected in the one participant meeting criteria for resistance testing. Virological suppression was achieved by 29/35 (83%, 95% CI 66-93%) with resistance to tenofovir and XTC, 11/13 (85%, 95% CI 55-98%) with resistance to XTC, and 6/6 (100%, 95% CI 54-100%) with resistance to neither.

**Conclusion:**

A high proportion of adults switching to second-line TLD achieved virologic suppression despite substantial baseline NRTI resistance and most not suppressed had low-level viraemia (≤100 copies/mL). This suggests recycling tenofovir and XTC with dolutegravir could provide an effective second-line option.

## Introduction

Until recently, boosted protease inhibitor (PI) regimens were the mainstay of second-line antiretroviral therapy (ART) in low and middle income settings. However, the DAWNING study demonstrated that in patients failing first-line non-nucleoside reverse transcriptase inhibitor (NNRTI)-based regimens, dolutegravir had superior efficacy and tolerability compared with a lopinavir-ritonavir regimen(1). On balance with concerns of neural tube defects and long-term weight gain, the World Health Organization (WHO) has since recommended dolutegravir for second-line ART in programmatic settings(2).

Dolutegravir is available in a fixed-dose combination with tenofovir and lamivudine (TLD) to virologically-suppressed patients on NNRTI-based first-line regimens. However, patients requiring second-line ART are not currently eligible for this treatment as most failed first-line regimens contain tenofovir and lamivudine/emtricitabine (XTC) as a backbone. A high proportion of patients failing first-line ART have nucleoside reverse transcriptase inhibitors (NRTI) resistance, particularly in sub-Saharan Africa(3,4), raising concern that recycling tenofovir and XTC from first to second-line could result in dolutegravir being the only fully active drug in the regimen, in turn risking the development of integrase resistance (which has been observed when dolutegravir is used as monotherapy(5,6)).

In DAWNING, at least one of the two NRTIs had to be fully active on resistance testing performed at screening. Resistance testing is not feasible at scale in lower-resource settings due to cost and laboratory capacity, so the WHO currently recommends switching patients failing a tenofovir-based first-line NNRTI-based regimen to zidovudine, lamivudine and dolutegravir(2) (thereby ensuring at least one fully active NRTI as tenofovir does not select for zidovudine resistance mutations(7)).

However, using zidovudine has disadvantages compared with tenofovir: it is more expensive, less well tolerated, has a greater pill burden and requires more frequent initial laboratory monitoring(8). TLD would thus be a more desirable second-line regimen than using zidovudine with dolutegravir, particularly in lower-resource settings.

Despite concerns regarding recycling tenofovir and XTC, compromised NRTIs have proven effective in PI regimens - several studies have demonstrated the virological efficacy of second-line ART that combines a ritonavir-boosted PI with two NRTIs even in the presence of resistance to both NRTIs(9–12). Dolutegravir’s high barrier to resistance and the cost to viral fitness of NRTI mutations(13) mean that recycling NRTIs with dolutegravir could also result in TLD providing an efficacious second-line regimen. Although there are clear advantages to patients and health services from such a strategy, there is currently insufficient evidence to recommend TLD in second-line regimens. We evaluated virological suppression at 24 weeks in a prospective cohort study of participants switched to TLD after failing a first-line regimen containing tenofovir, XTC and efavirenz or nevirapine.

## Methods

### Study design

We conducted a single arm, prospective, interventional study in two primary care clinics in Khayelitsha, a large, peri-urban informal settlement in Cape Town, South Africa, with nearly 50,000 patients accessing ART through the public health system. The protocol was approved by the University of Cape Town’s Human Research Ethics Committee (039/2019) and is available with the statistical analysis plan on ClinicalTrials.gov (NCT03991013).

### Participants and sample size

Eligible patients were HIV-positive adults who had failed a first-line regimen consisting of tenofovir, XTC and efavirenz or nevirapine, confirmed by two consecutive VL>1000 copies/mL 2-24 months apart (the most recent at screening). They were recruited from the primary care clinics through clinician referral or identification from folders. Exclusion criteria were: CD4<100 cells/μl, active or suspected tuberculosis, active AIDS-defining conditions, an estimated glomerular filtration rate<50 ml/min/1.73m^2^, haemoglobin<7.5 g/dL, alanine aminotransferase>100 IU/L, a previous or current diagnosis of malignancy or any condition judged to put the patient at increased risk if participating, a condition judged likely to impact adherence (active psychiatric disease or substance abuse), pregnancy, breastfeeding, intention to fall pregnant, or unable to take the study medication (allergy, intolerance or contra-indicated drug interaction). Women of child-bearing potential were required to be on effective contraception.

A sample size of 57 was calculated to produce a 95% confidence interval (CI) of 72-92%, with the assumption that VL suppression of 82% would be achieved in the modified intention-to-treat (mITT) analysis at week 24, as achieved by the dolutegravir arm at week 24 in the DAWNING study(1). To account for patients discontinuing the regimen we planned to enrol 65 participants. Due to COVID-19-related recruitment challenges and as no participants had been lost to follow-up by June 2020, the data safety monitoring committee allowed early completion of enrolment after 62 participants. We report on the outcomes of the first 60 participants who had completed 24 weeks follow-up.

### Procedures

After providing written informed consent, participants were switched from first-line ART to oral TLD (tenofovir 300mg, lamivudine 300mg, dolutegravir 50mg once daily). Participants received dolutegravir 50mg twice daily for the first 14 days because efavirenz induces enzymes and transporters involved with dolutegravir transport and metabolism, resulting in a marked reduction in dolutegravir exposure(14). The inducing effect of efavirenz is largely resolved two weeks after switching.

Study visits with clinicians occurred every four weeks to 24 weeks (with a ± two-week visit window at each time point, but extended to 6 weeks after the 24 week time-point to accommodate COVID restrictions). VL was assessed at baseline and every subsequent visit, with a repeat VL after two weeks of additional adherence counselling if VL was >50 copies/mL after week 12. If the repeat VL was >500 copies/mL a genotypic resistance test was performed. Baseline genotypic resistance testing, as previously described(15), was performed retrospectively for all participants and was not available to inform treatment decisions.

CD4 count was performed at baseline and 24 weeks, creatinine at baseline, four and 16 weeks and a pregnancy test at every visit for women of child-bearing potential. As insomnia, anxiety and depression are recognised side effects of dolutegravir, the insomnia severity index (ISI)(16) was conducted at each visit and mental health was assessed using the Modified Mini Screen (MMS)(17) at baseline, week 12 and week 24.

Tenofovir diphosphate (TFV-DP) concentrations on dried blood spots were conducted as an objective measure of adherence at week 0, 12 and 24 (batched and analysed after 24 weeks). An indirect method for the quantification of TFV-DP in 50 μl human dried blood spots was adapted from the method developed by Castillo-Mancilla et al(18) and validated at the Division of Clinical Pharmacology, University of Cape Town. It consisted of solid phase separation of tenofovir and TFV-DP, enzyme dephosphorylation of TFV-DP to tenofovir, followed by high performance liquid chromatography with tandem mass spectrometry detection of tenofovir(19).

During the COVID-19 pandemic national lockdown during April and May 2020, visits were rescheduled, participants given multi-month refills and follow-up conducted telephonically.

### Outcomes and analysis

The primary outcome was VL suppression (defined as VL <50 copies/mL) at week 24, evaluated using a mITT analysis, according to the FDA snapshot algorithm(20). We regarded the following as failure: those with measured VL ≥50 copies/ml, missing VL within the visit window, intolerance or adverse event due to any drug in the regimen requiring switch, and drug substitution not permitted by the protocol. Loss to follow-up and stopping or switching due to dolutegravir or NRTI intolerance or adverse events was regarded as failure. Switching due to stopping contraception, wish to become pregnant, becoming pregnant, transfer out for non-clinical reasons and death from non-HIV and non-drug causes (as assessed by the study doctor) were not regarded as failure.

Secondary outcomes included VL suppression at week 12, proportion suppressed over time, time to suppression, development of new resistance mutations, adherence (determined by TFV-DP concentration), and safety (creatinine, mental health and sleep assessments, pregnancy, adverse events, and mortality).

Categorical variables were described using proportions and continuous variables using median and interquartile range. Proportions were presented with the corresponding exact binomial 95% confidence interval. If the success proportion was 0 or 100%, a one-sided 97.5% confidence interval was estimated. Time-to-event endpoints were analysed using survival analysis. A pre-specified secondary analysis described VL suppression defined as <400 copies/mL. A pre-specified sensitivity analysis of VL suppression at 12 and 24 weeks was conducted excluding certain participants included in the mITT analysis: those lost to follow-up or missing a VL in the window, those with evidence of poor adherence at the visit (TFV-DP <350 fmol/punch) and those who stopped or were changed from the study drug for reasons other than treatment failure.

### Definitions

Virological failure was defined as having two consecutive VL >1000 copies/mL after week 12. Genotypic resistance was classified using the Stanford algorithm (version 8.9-1), with a score ≥15 indicating at least low-level resistance. Results were categorised as two fully active NRTIs (both with a Stanford score <15), resistance to one NRTI (one with a Stanford score <15 and one ≥15) and dual resistance to both NRTIs (both with a Stanford score ≥15)(21).

Adverse events were graded according to Division of AIDS (DAIDS) criteria(22). TFV-DP concentrations were categorised using the thresholds defined by Anderson et al(23) as <350 fmol/punch (men: <1.2 doses per week and women: <0.6 doses per week), 350-700 fmol/punch (men: 1.2-3.2 doses per week and women: 0.6-2.0 doses per week), 700-1250 fmol/punch (men: 3.2-6 doses per week and women: 2.0-5.3 doses per week) and > 1250 fmol/punch (men: >6 doses per week and women: >5.3 doses per week).

## Results

**Figure 1:**
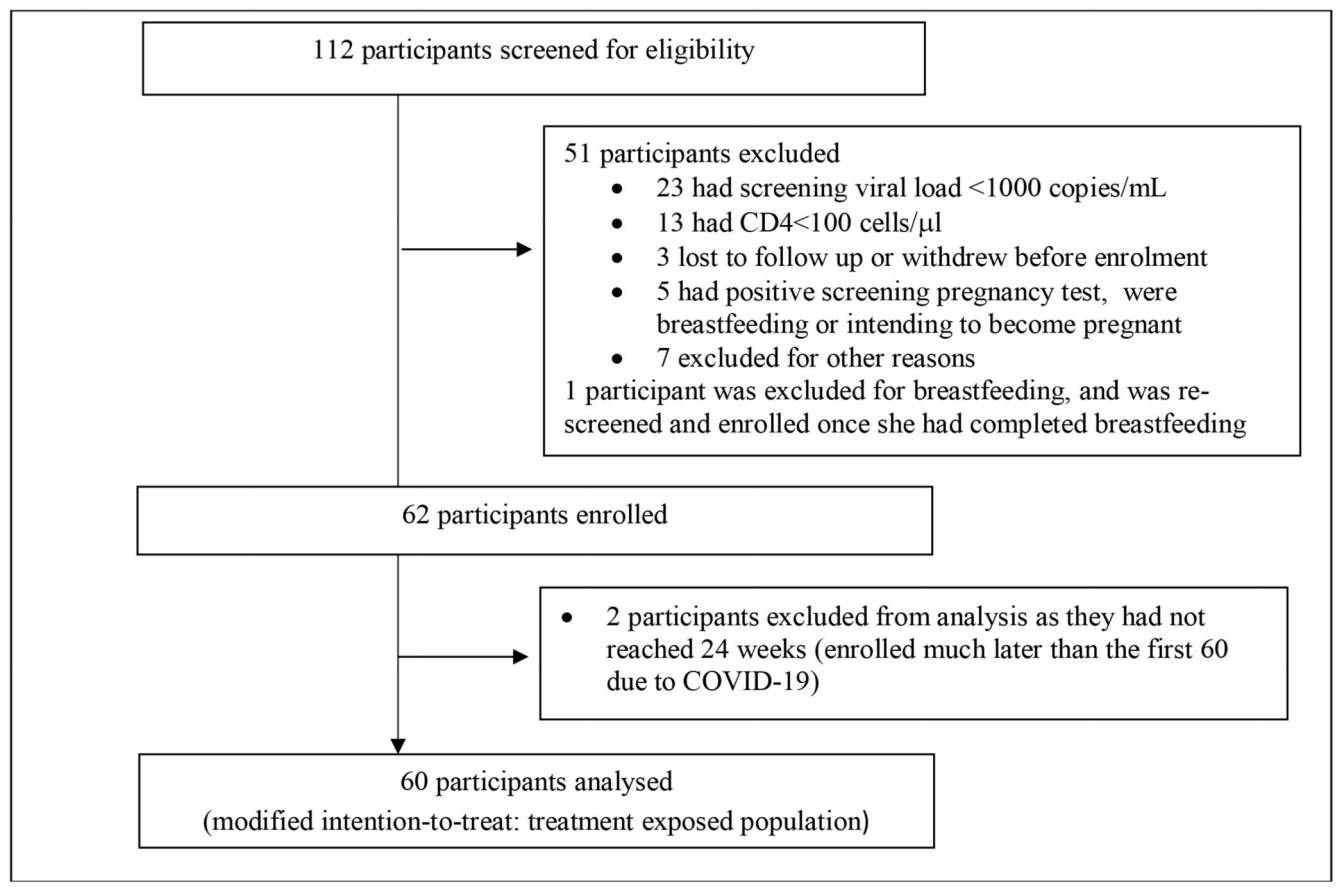
Study recruitment and enrolment

Between 8 August 2019 and 23 March 2020, 112 patients were screened and 62 participants were enrolled; 60 are included in this analysis (Figure 1).

The enrolled participants were mostly female (70%) and had a median of 5.8 years of experience with ART (IQR 2.8 to 8.3). The median VL was 10 580 copies/mL (IQR 2 962 to 38 291) and one participant had a VL greater than 100 000 copies/mL. Baseline resistance results were available for 54/60 participants (six samples failed sequencing) and 89% had at least low-level resistance to tenofovir or XTC: 20/54 (37%) participants had K65R and 45/54 (83%) had M184V/I (see Table 1 for baseline characteristics). Additionally, 19/54 (35%) had thymidine analogue mutations (Supplementary Table 1a and 1b).

### Viral load outcomes

At week 24, 51/60 (85%, 95% CI 73.4-92.9%) participants achieved virologic suppression in the mITT analysis (Table 2). The outcomes for the other nine participants were: six had a VL 50-99 copies/mL, one had a VL 100-999 copies/mL, one had switched study drug due to a tenofovir-related adverse event, and one did not have a VL performed in the window (Supplementary Table 2). One participant in the 50-99 copies/mL category had a VL reported as “<100 copies/mL” by the laboratory due to low volume sample, which limited the assay range. If this participant is reclassified as suppressed, suppression would be 52/60 (87%, 95% CI 75.4-94.1%).

**Figure 2:**
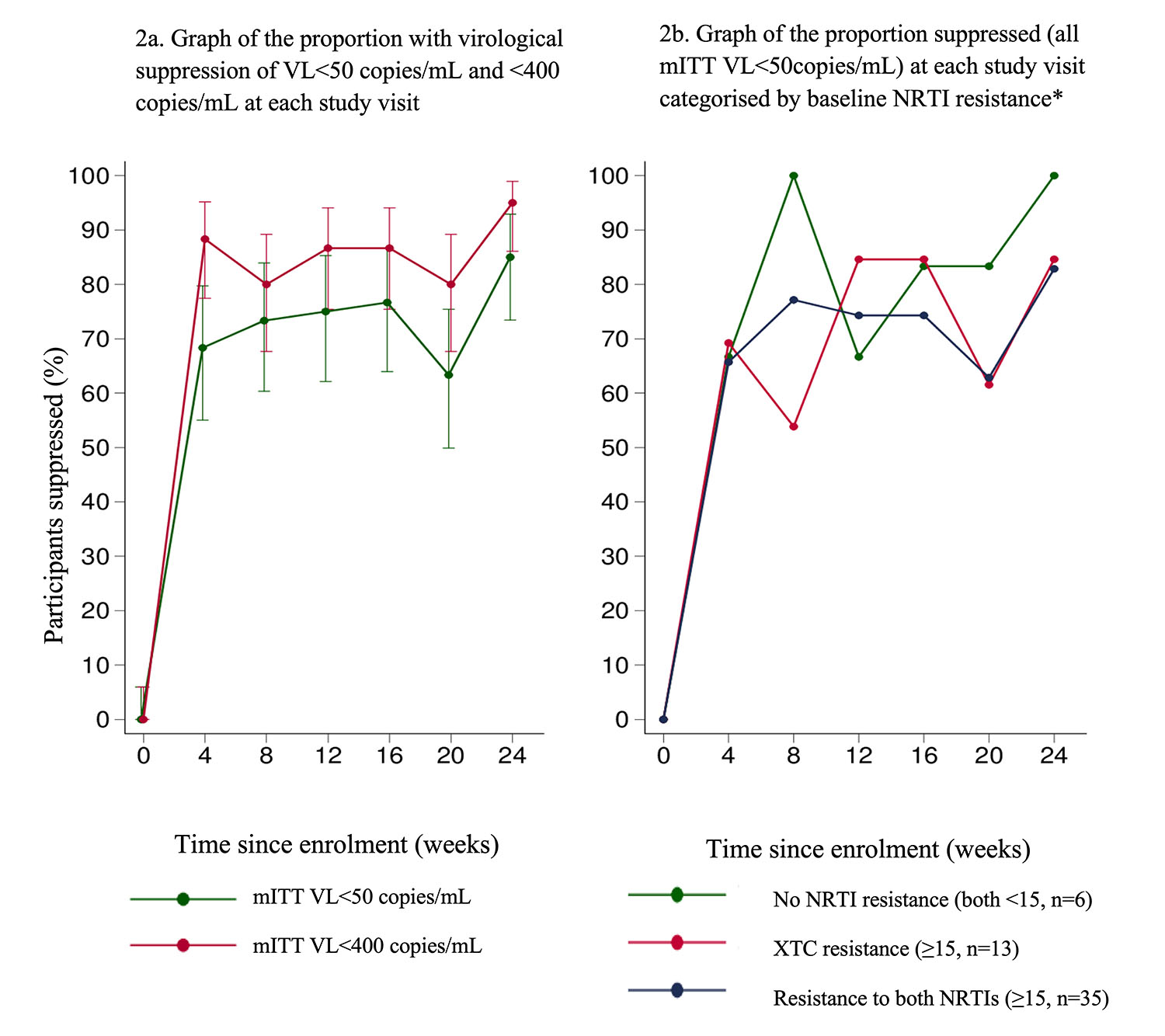
Proportion suppressed at each study visit in the modified intention-to-treat (mITT) NRTI (nucleoside reverse transcriptase inhibitor), VL (viral load), XTC (lamivudine or emtricitabine) * Genotypic resistance was classified using the Stanford algorithm (version 8.9-1), with a score ≥15 indicating at least low-level resistance. Results were categorised as 6/54 having 2 fully active NRTIs (both with a Stanford score <15), 13/54 with resistance to lamivudine (3TC) only (tenofovir with a Stanford score <15 and XTC with a Stanford score ≥15), 0/54 with resistance to tenofovir only and 35/54 with resistance to both NRTIs (both with a Stanford score ≥15)^7^

In a secondary mITT analysis defining VL suppression as <400 copies/mL, 57/60 (95%, 95% CI 86.1-99.0%) were suppressed at week 24 (Table 2). VL suppression at each visit is illustrated in Figure 2 and more granular VL results are presented in Supplementary Tables 3 and 4. VL suppression dropped at week 20 as 11 participants missed this visit due to COVID-19 lockdown restrictions.

The pre-specified sensitivity analysis at week 24 included 57 participants (three participants were removed from analysis, one for each of: missing a VL within the ±14-day window, low TFV-DP concentration <350 fmol/punch, and switched due to an adverse event), and showed 51/57 participants had a VL<50 copies/mL (89.5%, 95% CI 78.5-96.0%) and 57/57 participants had a VL<400 copies/mL (100%, 95% CI 93.7-100.0%) (Table 2).

Median time to suppression <50 copies/mL was 4.0 weeks (95% CI 4.0-4.9), at which point 41 participants (68.3%) were suppressed (Supplementary Figure 1).

No participants had study-defined virological failure (two consecutive VL >1000 copies/mL) by week 24. One participant (who reported periods of non-adherence and had a TFV-DP concentration <350 fmol/punch at week 24) had two consecutive VL >500 copies/mL and had genotypic resistance testing as per protocol: there were no integrase-inhibitor or NRTI resistance mutations, but NNRTI resistance mutations were detected (K103N and P225H). The baseline resistance sample for this participant failed sequencing.

Participant characteristics were mostly similar in those who suppressed at week 24 and those who did not. However, baseline VL was lower (8 320 copies/mL [IQR 2 608-24 971] vs 40 761copies/mL [IQR 22 197-56 219]) and duration on ART was longer (6.4 years [IQR 3.0-8.5] vs 3.4 years [IQR 1.2-6.3]) in those who suppressed <50 copies/mL, with similar findings for those who suppressed <400 copies/mL (Supplementary Tables 5 and 6). There was no statistically significant difference between TFV-DP concentrations in those who had a suppressed VL at week 24 compared with those who did not (n=49, Fisher’s exact p=0.3, Supplementary Table 5).

Suppression was similar when stratified by baseline age, sex, VL, CD4 count and ART history for both the primary endpoint and defining suppression as VL<400 copies/mL (Supplementary Figures 2 and 3). Virological suppression at week 24 was achieved by 29/35 (83%, 95% CI 66.4-93.4%) with resistance to tenofovir and XTC at baseline, 11/13 (85%, 95% CI 54.6-98.1%) of those with resistance to XTC only and 6/6 (100%, 95% CI 54,1-100.0%) of those with no NRTI resistance (Fisher’s exact p-value overall = 0.85, Supplementary Figure 2).

### Other outcomes at week 24

**Figure 3:**
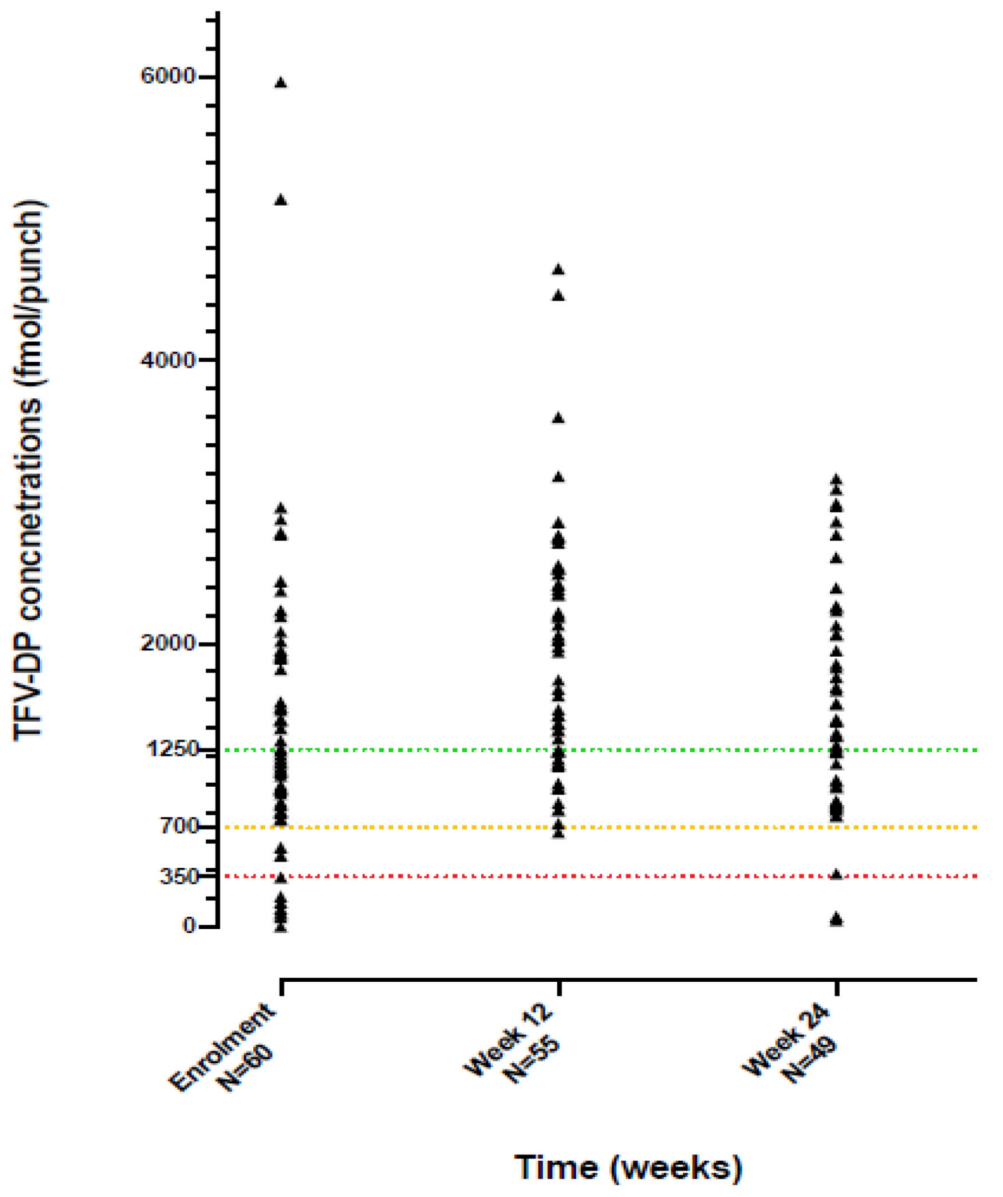
Tenofovir diphosphate (TFV-DP) dried blood spot concentrations at baseline, week 12 and week 24 TFV-DP concentration, used as a marker of adherence, was categorised using the thresholds defined by Anderson et al^23^ as: <350 fmol/punch (equivalent of men: <1.2 doses per week and women: <0.6 doses per week)350-700 fmol/punch (men: 1.2 -3.2 doses per week and women: 0.6 -2.0 doses per week)700-1250 fmol/punch (men: 3.2-6 doses per week and women: 2.0-5.3 doses per week)1250 fmol/punch (men: >6 doses per week and women: >5.3 doses per week)

At week 24, the median CD4 count was 373 cells/μl (IQR 237-501), a median increase from baseline of 99 cells/μl (IQR 36-163). The median increase in BMI was 0.8 kg/m^2^ (IQR 0.0-2.2) over 24 weeks, to 28.4 kg/m^2^ (IQR 23.1-34.0) and the median increase in weight was 2.2kg (IQR -0.1-6.2) to 74.8kg (IQR 64.7-83.9) at 24 weeks. The median TFV-DP concentration was 1157 fmol/punch at baseline, 1939 fmol/punch at week 12 and 1350 fmol/punch at week 24 (Figure 3 and Supplementary Table 7).

### Safety and tolerability outcomes

TLD was generally well tolerated with 7/60 (12%) of participants experiencing a DAIDS grade 3 or 4 adverse event and 4/60 (7%) experiencing a serious adverse event over 24 weeks (Supplementary Table 8). None of these were attributed to study drug and no participants died or became pregnant. One participant was switched from tenofovir to zidovudine after developing a creatinine elevation. No participants developed new or worsened scores indicating a DSM diagnosis of anxiety, mood or psychotic disorder on the MMS score, or indicating insomnia on the ISI (Supplementary Figures 4 and 5). One participant was treated with rifampicin (for incident tuberculosis); dolutegravir was increased to 50mg twice daily until two weeks after competing rifampicin as per protocol (Supplementary Table 9).

## Discussion

Ours is the first prospective, interventional study to evaluate the recycling of tenofovir and XTC with dolutegravir in second-line ART, and demonstrated that a high proportion of participants achieved virological suppression by week 24. Our study was embedded in the primary healthcare system in an urban informal settlement in South Africa, representing real-world patient conditions. Our findings provide preliminary evidence that patients failing a first-line ART regimen containing tenofovir, XTC and efavirenz or nevirapine, can be switched to TLD as second-line ART.

We found that 85% of participants achieved a VL<50 copies/mL at week 24, comparable to the 82% virologically suppressed at week 24 in the dolutegravir arm of the DAWNING study of second-line dolutegravir and an optimised NRTI backbone selected using resistance testing(1). With a VL threshold of 400 copies/mL, commonly used in programmatic settings, 95% of participants were suppressed at week 24. None of our participants developed virological failure by week 24; only one participant met criteria for resistance testing, which detected no integrase resistance mutations and the patient reported poor adherence, corroborated by the TFV-DP concentration <350 fmol/punch at week 24. Resistance to both tenofovir and lamivudine was present in 65% of our participants at baseline, with only 11% of participants having two fully active NRTIs. Baseline NRTI resistance did not appear to affect the likelihood of virological suppression by week 24, but we were under-powered for this analysis.

It is well-described that boosted-PIs are effective with compromised NRTIs(9–12). Our study builds the evidence base that dolutegravir might also be effective in second-line ART despite resistance to all NRTIs accompanying it. The ART-PRO study reported that no participants experienced virological failure when integrase-inhibitor-naïve, virologically suppressed patients were switched to dual therapy with dolutegravir plus lamivudine, even though 21 of the 41 participants had lamivudine resistance mutations in historical plasma genotypes(24). Similar findings were reported in switch studies evaluating lamivudine and dolutegravir dual therapy(25) and dolutegravir with companion drugs to which resistance was documented(26). The efficacy of TLD in our study, despite resistance to one or both of tenofovir and lamivudine in 89% of our participants, may be attributable to the crippling effect that NRTI mutations have on viral fitness, resulting in residual antiviral activity(13). In addition, there is in vitro evidence that K65R and M184V/I mutations may protect against the development of the key dolutegravir resistance mutation R263K(27).

The median time to suppression was four weeks, at which point 68% of participants were suppressed, similar to the 66% of second-line participants on dolutegravir with an optimised NRTI backbone who suppressed at week four in DAWNING(1). All of our participants who were not suppressed at week 24 had low level viraemia (most of them <100 copies/mL). None of those who did have a VL >50 copies/mL at any visit after week 12 developed virological failure and most re-suppressed with enhanced adherence counselling, which has repeatedly been shown to result in high rates of re-suppression(28–30). This is important to consider in interpreting whether unsuppressed viral loads represent the risk of failure due to resistance or rather poor adherence without development of resistance. While sustained low level viraemia can lead to accumulation of resistance mutations(31), virological failure is less likely to develop when the unsuppressed VL is <200 copies/mL than at higher VLs(32). In the ADVANCE study, low-level viraemia at the primary endpoint visit did not predict later virological failure in the dolutegravir arms, leading the authors to suggest that the endpoints for ART trials may need to be rethought in the era of second generation integrase inhibitors, which are more robust to the development of resistance(30).

Our study has limitations. Firstly, our sample size was small and we had no control arm. We had sufficient precision for our primary outcome, but not for the important secondary outcome, as discussed in DAWNING, of virologic response stratified by level of baseline NRTI resistance mutations. Secondly, as the development of integrase-inhibitor resistance may be delayed and occur 24 to 48 weeks after switching to dolutegravir(5,6), we will continue to monitor our participants until 96 weeks. Thirdly, all our participants had supplemental dolutegravir doses for two weeks to overcome efavirenz induction effects, and our findings may not be generalisable to patients not given supplemental doses.

It is unknown whether this supplementary dosing strategy is necessary. Efavirenz induces enzymes and transporters involved in dolutegravir absorption and metabolism, reducing plasma dolutegravir concentrations at the end of the dosing interval up to 75%(14,33). A pharmacokinetic sub-study of STRIIVING concluded that no dolutegravir dose adjustment is required when switching from efavirenz to dolutegravir, but this trial was conducted in virologically suppressed individuals in whom efavirenz was likely still active(34). Because all patients entered our study with elevated VLs and would likely have efavirenz resistance, we elected to include a two-week supplementary dose of dolutegravir (50mg twice daily). This dosing strategy has not been practiced in other second-line trials where small numbers of participants developed integrase-inhibitor resistance(35,36) or in low-income settings where infrequent cases of dolutegravir resistance have been reported as TLD has been introduced(37). We will be exploring this strategy in stage two of our study, in which patients will be randomised to supplementary dolutegravir 50mg or placebo for the first two weeks of second-line TLD.

Our findings that TLD was an efficacious second-line regimen and that baseline NRTI resistance did not impact virological suppression need to be confirmed in larger studies. This has significant implications for regimen choices as the aging HIV cohort gains experience on multiple ART regimens and pre-treatment resistance rates increase(38). Two large studies (Dolutegravir and Darunavir Evaluation in Adults Failing Therapy (D^2^EFT)(39) and Nucleosides and Darunavir/Dolutegravir in Africa (NADIA)(40)) are addressing the question of recycling NRTIs with dolutegravir in second-line – if these studies confirm our findings there would be important implications for switching patients to dolutegravir-based regimens, particularly in low-resource settings with limited access to viral load testing or single dose dolutegravir. Confirmation of our findings would suggest that all patients on first-line ART could be switched to TLD without first needing to perform a VL to confirm suppression, estimated to greatly simplify implementation and avert more DALYs than other more complex policy options that include performing a VL before switch(41). This model could show even more benefit if the emergence of dolutegravir resistance on second-line TLD is confirmed to be rare in larger trials, which our study suggests.

Our study demonstrated that a high proportion of patients failing first-line ART comprising tenofovir, XTC and efavirenz or nevirapine achieved virological suppression at week 24 on TLD, despite the presence of resistance to the recycled NRTIs in the majority of patients. Most of the participants who did not suppress had low-level viraemia, which may represent adherence rather than resistance issues. Our findings, if confirmed in larger ongoing controlled trials, create the opportunity to use TLD as a cheap, tolerable, single pill, second-line regimen. The switch of all patients on NNRTI-based regimens to TLD, regardless of VL, would also reduce costs associated with second-line treatment and monitoring, and simplify the implementation of TLD rollout in ART programmes globally.

## Supplementary Material

Supplementary material

## Figures and Tables

**Table 1:** Baseline characteristics (n=60)

Female sex (n/%)	42 (70%)
Age in years (median/IQR)	37 (31-46)
BMI in kg/m^2^ (median/IQR)	27.6 (23.4-32.5)
Weight in kg (median/IQR)	70 (62.3-81.2)
CD4 count in cells/μl (median/IQR)	248 (175-346)
VL in copies/mL (median/IQR)	10 580 (2 962-38 291)
NRTI genotypic resistance* (n/%) Two fully active NRTIs Resistance to one NRTI ○ Tenofovir, not XTC ○ XTC, not tenofovir Resistance to both NRTIs	6/54 (11%) 13/54 (24%) 0/54 (0%) 13/54 (24%) 35/54 (65%)
Efavirenz and/or nevirapine genotypic resistance* (n/%)	52/54 (96%)
ART history Duration of previous ART in years (median/IQR) Efavirenz at enrolment (n/%) Ever previously on zidovudine or stavudine as first-line ART (n/%)	5.8 (2.8-8.3) 59 (98%) 10 (17%)

*ART (antiretroviral therapy), BMI (body mass index), IQR (inter-quartile range), NRTI (nucleoside reverse transcriptase inhibitor), VL (viral load), XTC (lamivudine or emtricitabine)*

* Resistance classified using Stanford score ≥15 for that drug. Stanford score <15 indicates susceptible or potential low-level resistance to a drug, and ≥15 indicates low-level, intermediate, or high-level resistance to a drug^7^

**Table 2:** Viral load outcomes at week 12 and 24

	VL suppression <50copies/mL (n; percentage, 95% CI)		VL suppression <400copies/mL (n; percentage, 95% CI)	
	mITT analysis*	Sensitivity analysis**	mITT analysis*	Sensitivity analysis**
Week 12	45/60; 75.0 (62.1-85.3)	45/54; 83.3 (70.7-92.1)	52/60; 86.7 (75.4-94.1)	52/54; 96.3 (87.3-99.5)
**Week 24**	**51/60;** **85.0 (73.4-92.9)**	**51/57;** **89.5 (78.5-96.0)**	**57/60;** **95.0 (86.1-99.0)**	**57/57;** **100.0 (93.7-100.0)** †

* Modified intention-to-treat analysis (mITT) excludes those switching study drug for reasons of stopping contraception or wish to become pregnant, or becoming pregnant, transfer out for non-clinical reasons and death from non-HIV and non-drug causes.

** Sensitivity analysis excludes those excluded from mITT analysis, as well as lost to follow up, those missing a VL within the window, participants who stopped or were changed from the study drug for reasons other than failure of the regimen and those who had evidence of poor adherence (TFV-DP<350 fmol/punch).

† One sided 97.5% confidence interval (CI)
