# Supplementary material for: Virologic efficacy of tenofovir, lamivudine and dolutegravir as second-line antiretroviral therapy in adults failing a tenofovir-based first-line regimen: a prospective cohort study
Source: AIDS. Author manuscript; Available in PMC 2021 Nov 24. (PMC7612028; doi:10.1097/QAD.0000000000002936)
Supplement: Supplementary material [file EMS124473-supplement-Supplementary_material.pdf]

Supplementary material

This appendix is a supplement to: Keene et al. ***Virologic efficacy of tenofovir, lamivudine and dolutegravir as second-line in adults failing a tenofovir-based first-line regimen: a prospective cohort study***

Table 1a. Frequency of specific major nucleoside reverse-transcriptase inhibitor (NRTI) and non-nucleoside reverse-transcriptase inhibitor (NNRTI) mutations at baseline

Table 1b. Listing of major and minor nucleoside reverse-transcriptase inhibitor (NRTI) and non-nucleoside reverse-transcriptase inhibitor (NNRTI) mutations at baseline by participant

Table 2. Outcomes for participants not suppressed (viral load [VL] <50 copies/mL) using the modified intention-to-treat analysis at week 24

Table 3. Aggregated HIV viral load outcomes by study visit

Table 4. HIV viral load data by participant

Figure 1. Time to virologic suppression <50 copies/mL by modified intention-to-treat analysis

Table 5. Characteristics by primary outcome (viral load <50 copies/mL) at week 24 (modified intention-to-treat analysis, n=60)

Table 6. Characteristics by viral load <400 copies/mL at week 24 (modified intention-to-treat analysis, n=60)

Figure 2. Subgroup analysis of primary endpoint stratified by baseline characteristics and adherence (tenofovir diphosphate [TFV-DP] concentration) at week 24 (VL < 50 copies/mL at week 24, modified intention-to-treat analysis)

Figure 3. Subgroup analysis of participants with viral load <400 copies/mL at week 24 stratified by baseline characteristics and adherence (tenofovir diphosphate concentration) at week 24 (modified intention-to-treat analysis)

Table 7. Categorised tenofovir diphosphate (TFV-DP) concentrations at baseline, week 12, and week 24, stratified by sex

Table 8. Summary of adverse events from baseline to week 24

Figure 4. Proportion of individuals with specific Modified Mini Screen (MMS) score over time (continuous score ranging from 0 to 22)

Figure 5. Proportion of individuals with insomnia severity index (ISI) score at each timepoint (continuous measure ranging from 0 to 28)

Table 9.3 Concomitant medications with potential for dolutegravir interaction taken at any point from baseline to week 24

Table 1a. Frequency of specific major nucleoside reverse-transcriptase inhibitor (NRTI) and non-nucleoside reverse-transcriptase inhibitor (NNRTI) mutations at baseline

**Major NRTI mutations (n/%)**	**N=54**	**Major NNRTI mutations (n/%)**	**N=54**
M41L	2 (4)	L100I	2 (4)
K65R	20 (37)	K101E	5 (9)
K65N	0 (0)	K101P	0 (0)
K65E	0 (0)	K103H	0 (0)
D67N	10 (19)	K103N	31 (57)
T69Ins	0 (0)	K103S	1 (2)
T69Deletion	1 (2)	K103T	0 (0)
K70E	4 (7)	V106A	0 (0)
K70G	0 (0)	V106M	17 (31)
K70N	0 (0)	E138K	0 (0)
K70Q	2 (4)	Y181C	6 (11)
K70R	7 (13)	Y181F	0 (0)
K70S	0 (0)	Y181G	0 (0)
K70T	2 (4)	Y181I	0 (0)
L74I	2 (4)	Y181S	0 (0)
Y115F	3 (6)	Y181V	0 (0)
Q151L	0 (0)	Y188C	1 (2)
Q151M	0 (0)	Y188F	0 (0)
M184V	45 (83)	Y188H	0 (0)
M184I	0 (0)	Y188L	8 (15)
L210W	0 (0)	G190A	6 (11)
T215F	0 (0)	G190C	0 (0)
T215Y	0 (0)	G190E	1 (2)
		G190Q	0 (0)
		G190S	1 (2)
		G190T	0 (0)
		G190V	0 (0)
		P225H	16 (30)
		F227C	0 (0)
		M230L	1 (2)
Major mutations as defined by the Stanford university resistance database (last updated 2019-07-31)[3]			

Table 1b. Listing of major and minor nucleoside reverse-transcriptase inhibitor (NRTI) and non-nucleoside reverse-transcriptase inhibitor (NNRTI) mutations at baseline by participant

NO	NRTI, MAJOR	NRTI, MINOR	NNRTI, MAJOR	NNRTI, MINOR
1	Y115F,M184V,K65R		Y181C	V108I
2	D67N,K70R,M184V	K219E	V106M	F227L
3			K103N	
4	M184V,L74I	K219Q	P225H,K103N	A98G
5	K65R,M184V	V75I	K103N,L100I	
6	M184V,K65R		K103N,Y188L	
7	M184V,K65R	A62V,F77L,K219E	K103N,Y181C,G190A	
8	D67N,K70E,M184V		K103N,Y188L	H221Y
9	K70R,D67N,M184V	K219E,T215I	K103N	V108I,K238T
10	K70E,D67DN,M184V	K219KR	K103N,P225H	
11	M184V,K70Q		V106M,G190A,K101E	
12	K70T,M41L,K65R,M184V		V106M,Y188L	E138A
13	M184V,M41L,K65R,K70T		V106M,Y188L	E138A
14	M184V		K103N	V108I
15	K65R,M184V,Y115F,L74LI,D67N	K219E	V106M,K103N	
16	M184V	L74V	P225H,L100I,K103N	K238T
17	M184V,K70KR	K219E	V106M	V108I,F227FL
18	M184V,K70R,D67N	T215IV,K219E	Y188L	A98G
19	K70EQ,M184V		V106M	F227FL,E138A,V179D
20			K103N	
21	M184V			E138A
22	M184V,K70R,D67N	K219Q	V106M	F227L,E138Q
23	M184V,K65R	A62V	V106M,K103S	F227L
24	M184V		P225H,K103N	
25	D67N,M184V,K70E	K219R	K103N,P225H	H221Y,E138EG,F227FL,V108VI
26	M184V		Y188L,P225H	E138Q
27	K65R,M184V		K101E,Y181C,G190S	
28	M184V	V75VM	K103N,P225H	
29	D67N,K70R,M184V	K219E,T215I	G190E	
30	M41ML,M184V,K65R,L74L		K103N	E138EG,V108I
31	K65R	A62AV	Y181C,V106M	H221Y,F227L
32	M184V	A62AV	G190GA,P225H,K103N	K238KT
33	K65R,M184V	A62AV	V106M	V179D
34	M184V,Y115F,K65R		V106M,K103N	F227L
35	M184V,K65R		P225H,K103N	
36	K65R,M184V		V106M,M230L	V179D
37	K65R,M184V	A62V,K219E	K103N,G190A	
38			K103N,P225H	
39			K103N	
40	K65R,D67DN		Y181C,K101E,G190A,V106VM	

**41**			P225H,K103N	
**42**	M184V,K70KT,K65R	V75M,A62AV	Y188C,V106M	
**43**	M184V		P225H,K103N	V108I
**44**	M184V,K65R	A62V	Y188L,K103N	
**45**	D67N,K70R,M184V	K219Q	G190A,V106M,K101E	F227L
**46**	M184V,K70KE		K101KE,Y188L,P225PH	V106VI
**47**	M184V		Y181C,K101E,V106M,G190A	
**48**	L74I,M184V		K103N,P225H	A98G,H221Y,V108I
**49**	M184V		P225H,K103N	
**50**	K65R, T69Deletion	K219R,A62V	K103N,V106M	
**51**	M184V,K70Q		K103N	V108I,E138Q,L234LI
**52**	D67N,M184V,K70R	K219E	P225H,K103N	
**53**				
**54**	L74LI,M184V,K65KR,K70KN		K103N,P225H	

Major and minor mutations as defined by the Stanford university resistance database (last updated 2019-07-31)[3]

Table 2. Outcomes for participants not suppressed (viral load [VL] <50 copies/mL) using the modified intention-to-treat analysis at week 24

Outcome	N(%)	Details
VL >1000 copies/ml	0/60 (0%)	
VL 50-1000 copies/ml	7/60 (12%)	Six had a VL 50-99 copies/mL and one had a VL 100-999 copies/mL
Discontinued study drug (failure)	0/60 (0%)	
Discontinued study drug (other)	1/60 (1.7%)	Participant discontinued the study drug due to a tenofovir-related adverse event
Missing data but still on study	1/60 (1.7%)	Missed the study visit within the ±2 week VL window

Table 3. Aggregated HIV viral load outcomes by study visit

	Participants analysed (n)		Percentage suppressed (95% confidence interval)			
Week	mITT*	Sensitivity analysis**	<50cp/mL (mITT)	<50cp/mL (sensitivity)	<400cp/mL (mITT)	<400cp/mL (sensitivity)
0	60	60	0.0 (0.0-6.0)†	0.0 (0.0-6.0)†	0.0 (0.0-6.0)†	0.0 (0.0-6.0)†
4	60	54	68.3 (55.0-79.7)	75.9 (62.4-86.5)	88.3 (77.4-95.2)	98.1 (90.1-100.0)
8	60	51	73.3 (60.3-83.9)	86.3 (73.7-94.3)	80.0 (67.7-89.2)	94.1 (83.8-98.8)
**12**	**60**	**54**	**75.0 (62.1-85.3)**	**83.3 (70.7-92.1)**	**86.7 (75.4-94.1)**	**96.3 (87.3-99.5)**
16	60	52	76.7 (64.0-86.6)	88.5 (76.6-95.6)	86.7 (75.4-94.1)	100.0 (93.2-100.0)†
20	60	48	63.3 (49.9-75.4)	79.2 (65.0-89.5)	80.0 (67.7-89.2)	100.0 (92.6-100.0)†
**24**	**60**	**57**	**85.0 (73.4-92.9)**	**89.5 (78.5-96.0)**	**95.0 (86.1-99.0)**	**100.0 (93.7-100.0)†**

* Modified intention-to-treat analysis (mITT) excludes those switching study drug for reasons of stopping contraception or wish to become pregnant, or becoming pregnant, transfer out for non-clinical reasons and death from non-HIV and non-drug causes

** Sensitivity analysis excludes those excluded from mITT analysis, as well as lost to follow up, those missing a VL within the window, participants who stopped or were changed from the study drug for reasons other than failure of the regimen and those who had evidence of poor adherence (tenofovir diphosphate [TFV-DP] <350 fmol/punch). As TFV-DP was measured at weeks 12 and 24 only, it was assumed that individuals that were adherent/nonadherent at those visits were adherent/nonadherent respectively at the preceding visits (ie, weeks 4-12 for the week 12 adherence measure and weeks 16-24 for the week 24 adherence measure). Using this assumption, only one additional visit (at week 16) was excluded from the sensitivity population.

† One sided 97.5% confidence interval (CI) when 0 or 100% were successful

Table 4. HIV viral load (VL) data by participant

	VL result by week						
Participant	Screening VL	Week 4	Week 8	Week 12	Week 16	Week 20	Week 24
1	77425	314	215	69	102	58	49
2	4668	<20	LDL	29	LDL	23	LDL
3	2113	LDL	LDL	LDL	LDL	<20	<20
4	2608	33	<20	LDL	LDL	LDL	LDL
5	3097	LDL	<20	LDL	LDL	<100	<20
6	1299	26	31	80	39	50	<20
7	11178	<20	<20	70	22	MV	LDL
8	8320	<20	<20	LDL	24	<100	<20
9	17356	319	22467	<20	107	LDL	<20
10	61758	45	<20	<20	LDL	32	27
11	48765	73	<20	LDL	39	LDL	75
12	22197	77	<20	30	<20	30	52
13	1040	LDL	LDL	LDL	<20	LDL	LDL
14	44742	<100	LDL	LDL	MV	LDL	LDL
15	2218	<20	21	<20	LDL	37	81
16	22794	34	LDL	<20	<20	83	<100
17	79908	56	<20	24	40	<20	21
18	3323	LDL	LDL	LDL	<20	LDL	LDL
19	6104	89	62	193	112	109	53
20	37243	LDL	26	<20	LDL	LDL	LDL
21	6282	<20	<20	<20	LDL	LDL	LDL
22	3432	LDL	166	<20	LDL	LDL	<20
23	4662	<20	33	31	30	21	25
24	86446	71	<20	26	LDL	143	21
25	24971	1816	869	5300	67	97	<20
26	12649	23	<20	LDL	LDL	LDL	<50
27	7429	<20	21	<20	<20	<100	LDL
28	3163	<20	LDL	LDL	LDL	<20	LDL
29	25127	LDL	LDL	LDL	LDL	162	<20
30	18081	LDL	LDL	LDL	45	LDL	<20
31	16123	<20	20	LDL	LDL	LDL	LDL
32	12661	<20	<20	<20	LDL	MV	LDL
33	2138	29	34	<20	MV	MV	LDL
34	64216	41	68	105	74038	MV	755
35	22809	<20	<20	<20	38	MV	<20
36	20846	32	LDL	LDL	<20	MV	LDL

37	11414	<20	24	21	LDL	MV	27
38	1952	66	LDL	221	LDL	MV	LDL
39	1147	<20	34	<20	MV	MV	LDL
40	92677	<20	<20	MV	MV	<50	<20
41	7383	<20	<20	MV	132	22	LDL
42	7027	<20	34	1108	MV	<50	<50
43	39339	50	<20	MV	<20	<50	<20
44	18426	22	<20	MV	<20	LDL	LDL
45	9981	LDL	LDL	LDL	<20	MV	LDL
46	2061	LDL	MV	<20	<20	LDL	<20
47	9190	LDL	MV	LDL	LDL	LDL	<20
48	57190	LDL	MV	MV	<20	LDL	LDL*
49	1834	21	MV	<20	LDL	LDL	LDL
50	44757	<20	MV	<20	<20	MV	<20
51	124717	195	MV	35	33	<20	20
52	40761	68	MV	206	107	MV	MV
53	1009	LDL	MV	<20	LDL	<20	LDL
54	2827	MV	LDL	LDL	LDL	<20	21
55	6697	MV	MV	46	39	<20	<20
56	1923	MV	LDL	<50	27	44	26
57	54005	MV	<20	<50	<20	<20	<20
58	56219	MV	48	MV	LDL	32	63
59	1495	LDL	LDL	MV	LDL	LDL	LDL
60	2265	MV	3216	36	MV	<20	<50

Key	VL suppressed <50 copies/mL	VL 50-99 copies/mL	VL 100-999 copies/mL	VL ≥ 1000 copies/mL	MV: missed visit

Result considered suppressed if the value is <50 copies/mL, or noted in the report as "<50", "<20" or "LDL" (lower than detectable limit)

VL "<100": Based on the low sample volume, the linear range of this assay in dilution was 100 - 10,000,000 copies/mL.

* This participant was considered as a 'virological failure' in the modified intention-to-treat analysis as they were switched due to a tenofovir-related adverse event

Figure 1. Time to virologic suppression <50 copies/mL by modified intention-to-treat analysis

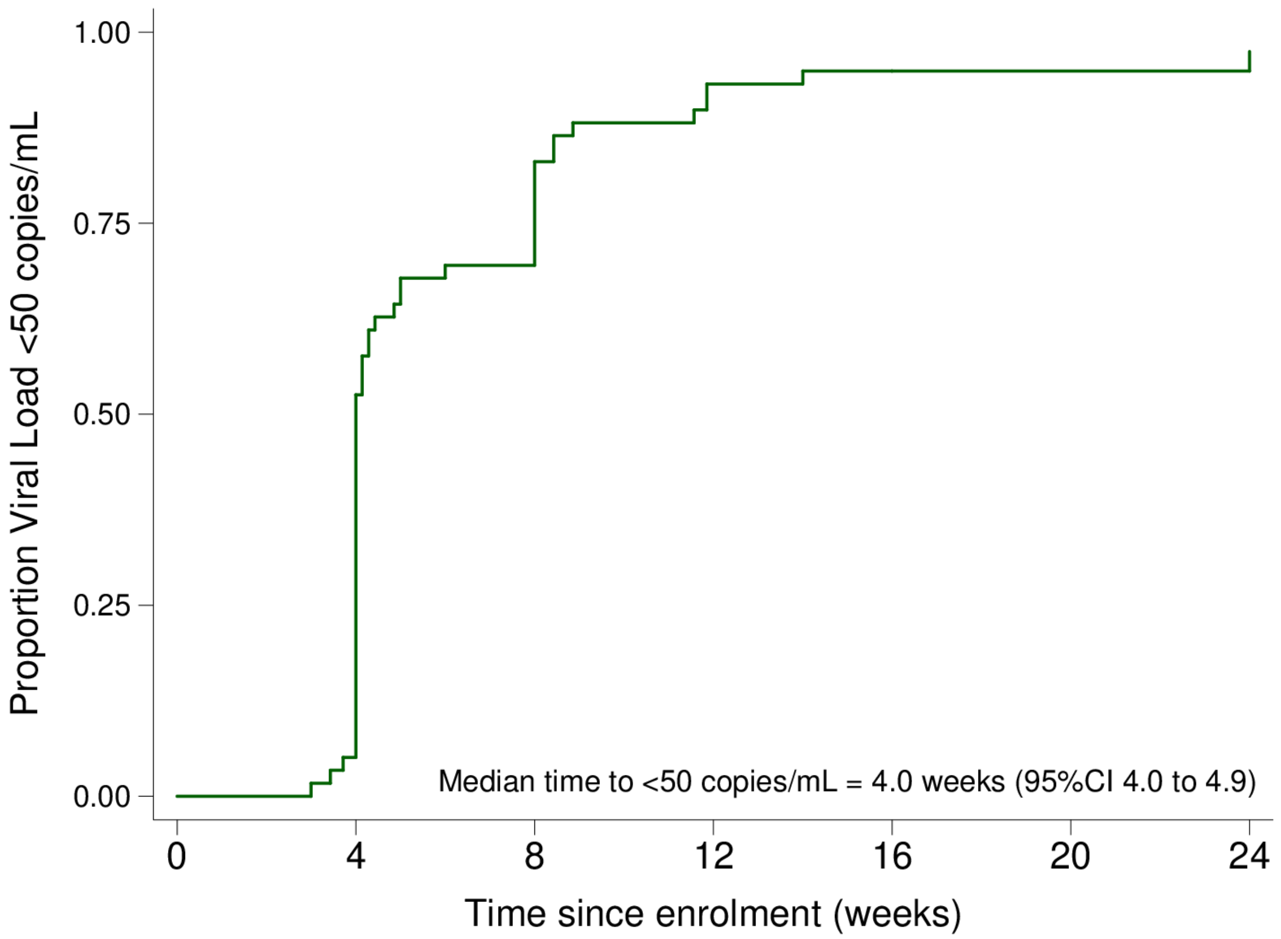

Table 5. Characteristics by primary outcome (viral load <50 copies/mL) at week 24 (modified intention-to-treat analysis, n=60)

	Success at week 24 (VL<50 copies/mL) (n=51)	Failure at week 24 (VL not <50 copies/mL) (n=9)
**Participant characteristics**		
Female, (n/%)	37 (73%)	5 (56%)
Age in years, (median/IQR)	37 (31-46)	36 (30-51)
**Baseline HIV characteristics**		
CD4 count in cells/mm3 (median/IQR)	294 (174-380)	202 (175-236)
Viral load in copies/mL (median/IQR)	8 320 (2 608-24 971)	40 761 (22 197-56 219)
**ART history**		
Previous ART duration in years (median/IQR)	6.4 (3.0-8.5)	3.4 (1.2-6.3)
Ever on zidovudine or stavudine (n/%)	9/51 (18%)	1/9 (11%)
**Baseline resistance*, n(%)**		
Tenofovir/ XTC resistance		
Both <15	6/46 (13%)	0/8 (0%)
Only tenofovir ≥15	0/46 (0%)	0/8 (0%)
Only XTC ≥15	11/46 (24%)	2/8 (25%)
Both ≥15	29/46 (63%)	6/8 (75%)
Efavirenz and/or nevirapine resistance	44/46 (96%)	8/8 (100%)
**TFV-DP concentrations at week 24 (fmol/punch)****		
<350, n (%)	1/42 (2%)	1/7 (14%)
350-700, n (%)	1/42 (2%)	0 /7 (0%)
701-1250, n (%)	17/42 (41%)	1/7 (14%)
>1250, n (%)	23/42 (55%)	5/7 (71%)

ART (antiretroviral therapy), VL (viral load), XTC (lamivudine or emtricitabine)

* Resistance classified using Stanford interpretation, where a score <15 indicates susceptible or potential low-level resistance to a drug, and ≥15 indicate low-level, intermediate, or high-level resistance to a drug[3]

** Tenofovir diphosphate (TFV-DP) concentration categorisation: <350 fmol/punch (equivalent of - men: <1.2 doses per week and women: <0.6 doses per week), 350-700 fmol/punch (men: 1.2 - 3.2 doses per week and women: 0.6 - 2.0 doses per week), 700-1250 fmol/punch (men: 3.2-6 doses per week and women: 2.0-5.3 doses per week) and > 1250 fmol/punch (men: >6 doses per week and women: >5.3 doses per week)[4].

Table 6. Characteristics by viral load <400 copies/mL at week 24 (modified intention-to-treat analysis, n=60)

	Success at week 24 (VL<400 copies/mL) (n=57)	Failure at week 24 (VL not<400 copies/mL) (n=3)
**Participant characteristics**		
Female, (n/%)	39 (68)	3 (100)
Age in years, (median/IQR)	37 (31-46)	34 (28-51)
**Baseline HIV characteristics**		
CD4 count in cells/mm3 (median/IQR)	263 (175-349)	189 (110-241)
Viral load in copies/mL (median/IQR)	9 190 (2 827-24 971)	57 190 (40 761-64 216)
**ART history**		
Previous ART duration in years (median/IQR)	6.3 (3.0-8.3)	2.7 (1.2-5.1)
Ever on zidovudine or stavudine (n/%)	10 (18)	0 (0)
**Baseline resistance*, n(%)**	(n=52)	(n=2)
Tenofovir/ XTC resistance		
Both <15	6 (12)	0 (0)
Only tenofovir ≥15	0 (0)	0 (0)
Only XTC ≥15	12 (23)	1 (50)
Both ≥15	34 (65)	1 (50)
Efavirenz and/or nevirapine resistance	50 (96)	2 (100)
**TFV-DP concentrations at week 24 (fmol/punch)****		
<350, n (%)	1/48 (4%)	1/1 (100%)
350-700, n (%)	1/48 (4%)	0/1 (0%)
701-1250, n (%)	18/48 (38%)	0/1 (0%)
>1250, n (%)	28/48 (58%)	0/1 (0%)

ART (antiretroviral therapy), VL (viral load), XTC (lamivudine or emtricitabine)

* Resistance classified using Stanford interpretation, where a score <15 indicates susceptible or potential low-level resistance to a drug, and ≥15 indicate low-level, intermediate, or high-level resistance to a drug[3]

** Tenofovir diphosphate (TFV-DP) concentration categorisation: <350 fmol/punch (equivalent of - men: <1.2 doses per week and women: <0.6 doses per week), 350-700 fmol/punch (men: 1.2 - 3.2 doses per week and women: 0.6 - 2.0 doses per week), 700-1250 fmol/punch (men: 3.2-6 doses per week and women: 2.0-5.3 doses per week) and > 1250 fmol/punch (men: >6 doses per week and women: >5.3 doses per week)[4].

Figure 2. Subgroup analysis of primary endpoint stratified by baseline characteristics and adherence (tenofovir diphosphate concentration) at week 24 (VL < 50 copies/mL at week 24, modified intention-to-treat analysis)

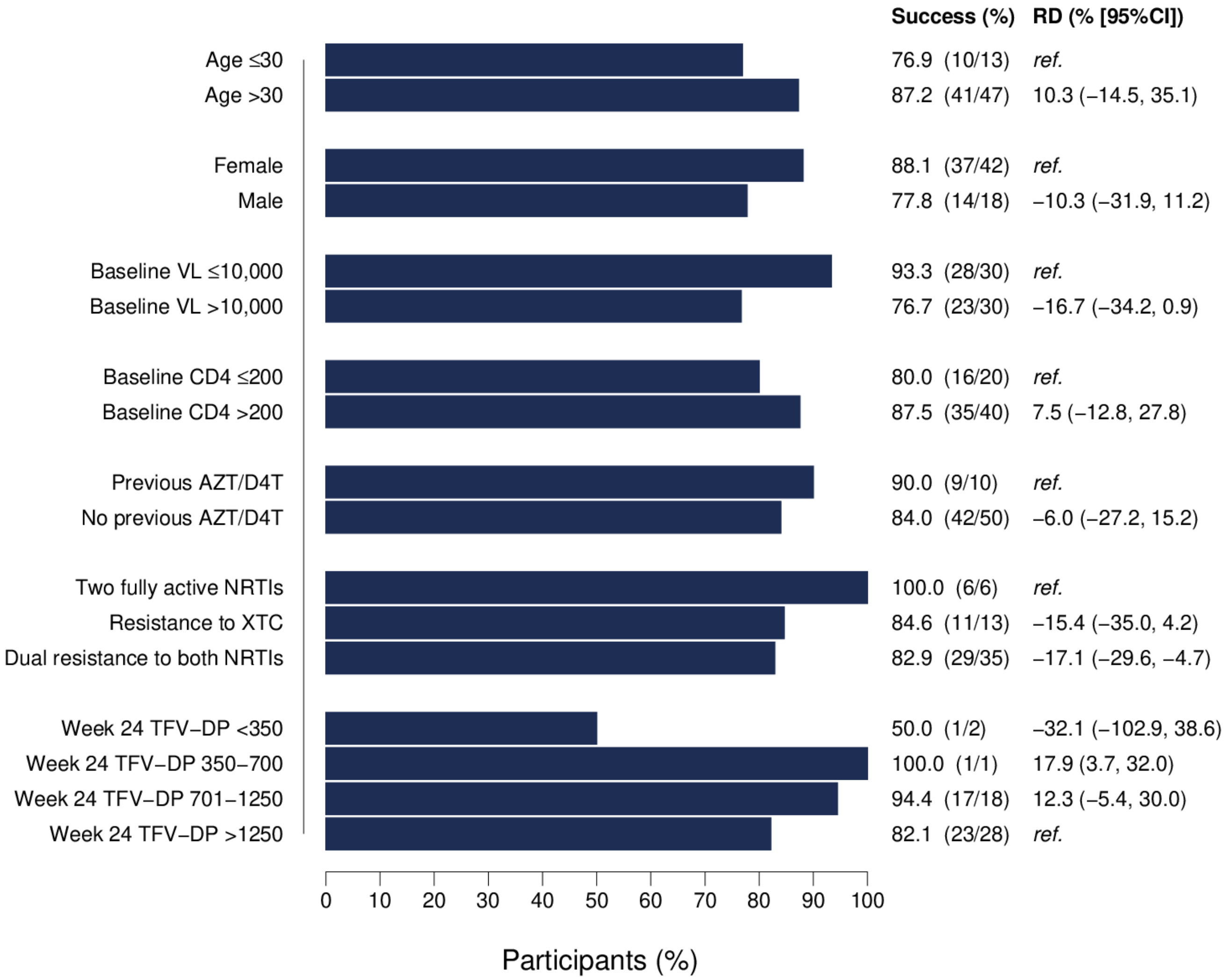

AZT (zidovudine), D4T (stavudine), NRTI (nucleoside reverse transcriptase inhibitor), TFV-DP (tenofovir diphosphate), *VL (viral load), XTC (lamivudine or emtricitabine)*

Figure 3. Subgroup analysis of participants with viral load <400 copies/mL at week 24 stratified by baseline characteristics and adherence (tenofovir diphosphate concentration) at week 24 (modified intention-to-treat analysis)

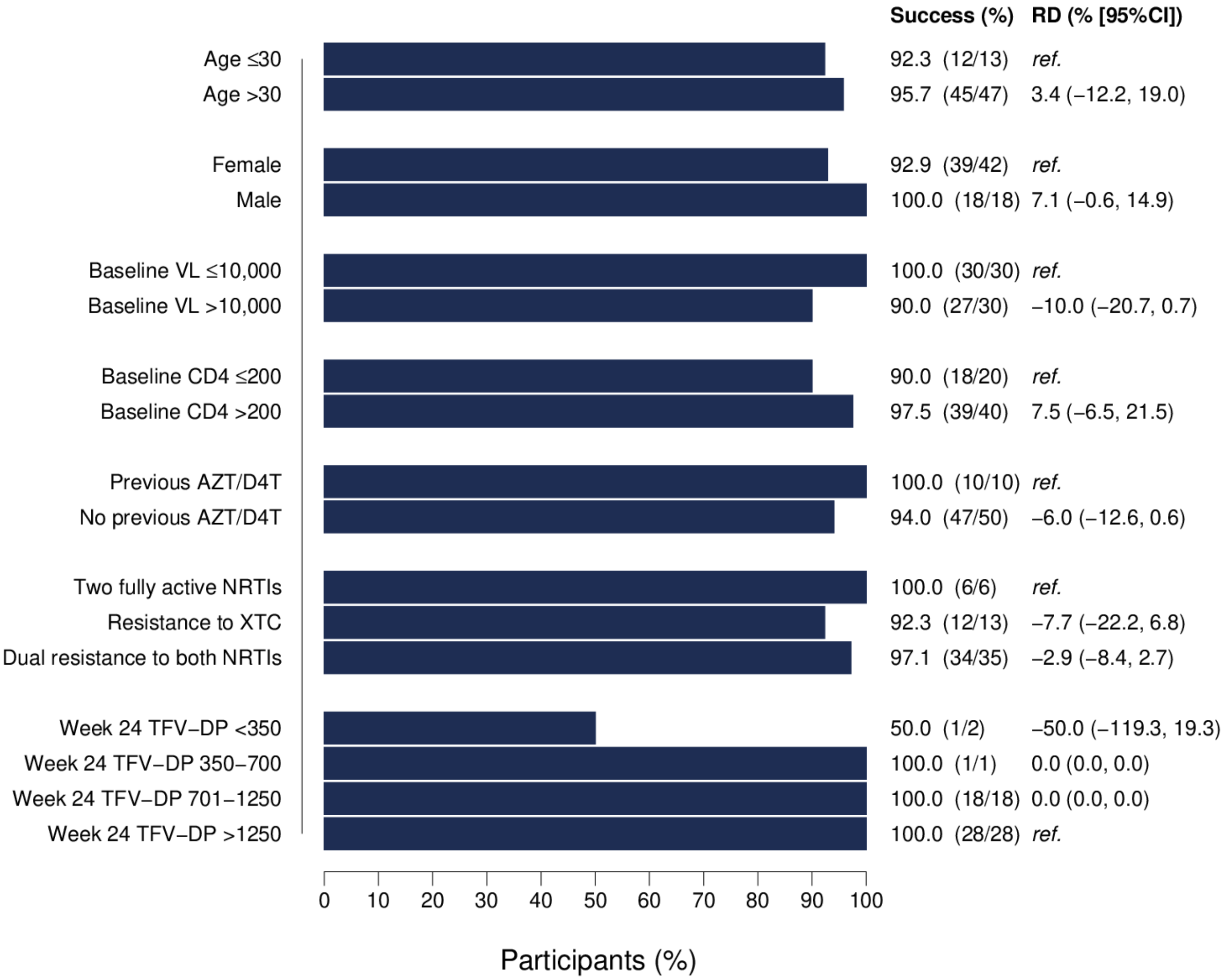

AZT (zidovudine), D4T (stavudine), NRTI (nucleoside reverse transcriptase inhibitor), TFV-DP (tenofovir diphosphate), *VL (viral load), XTC (lamivudine or emtricitabine)*

Table 7. Categorised tenofovir diphosphate (TFV-DP) concentrations at baseline, week 12, and week 24, stratified by sex

TFV-DP (fmol/punch)*	Baseline (N=60), n (%)		Week 12 (N=55), n (%)		Week 24 (N=49), n (%)	
	Women (N=42)	Men (N=18)	Women (N=38)	Men (N=17)	Women (N=34)	Men (N=15)
**<350, n (%)**	7 (16.7)	1 (5.6)	0	0	2 (5.9)	0
**350-700, n (%)**	0	2 (11.1)	1 (2.6)	0	1 (2.9)	0
**701-1250, n (%)**	18 (42.8)	6 (33.3)	11 (29)	4 (23.5)	14 (41.2)	4 (26.7)
**>1250, n (%)**	17 (40.5)	9 (50)	26 (68.4)	13 (76.5)	17 (50)	11 (73.3)

* TFV-DP concentration categorisation: <350 fmol/punch (equivalent of - men: <1.2 doses per week and women: <0.6 doses per week), 350-700 fmol/punch (men: 1.2 - 3.2 doses per week and women: 0.6 - 2.0 doses per week), 700-1250 fmol/punch (men: 3.2-6 doses per week and women: 2.0-5.3 doses per week) and > 1250 fmol/punch (men: >6 doses per week and women: >5.3 doses per week)[4].

Table 8. Summary of adverse events (AE) from baseline to week 24

	Number of individuals (n/%)	Total number of events
Mortality (all-cause)	0 (0%)	0
Serious AEs*	4 (7%)	4
Grade 3 and 4 AEs	7 (12%)	11
Clinical	5 (8%)	7
Grade 3	5 (8%)	7
Grade 4	0 (0%)	0
Laboratory	4 (7%)	4
Grade 3	3 (5%)	3
Grade 4	1 (2%)	1
Adverse events requiring discontinuation of any ART medication*	1 (2%)	1

* Serious AEs are defined as adverse events that result in death, a life-threatening incident, hospitalisation, disability, congenital abnormality or requires medical or surgical intervention to prevent permanent damage.

Figure 4. Proportion of individuals with specific Modified Mini Screen (MMS) score over time (continuous score ranging from 0 to 22*)

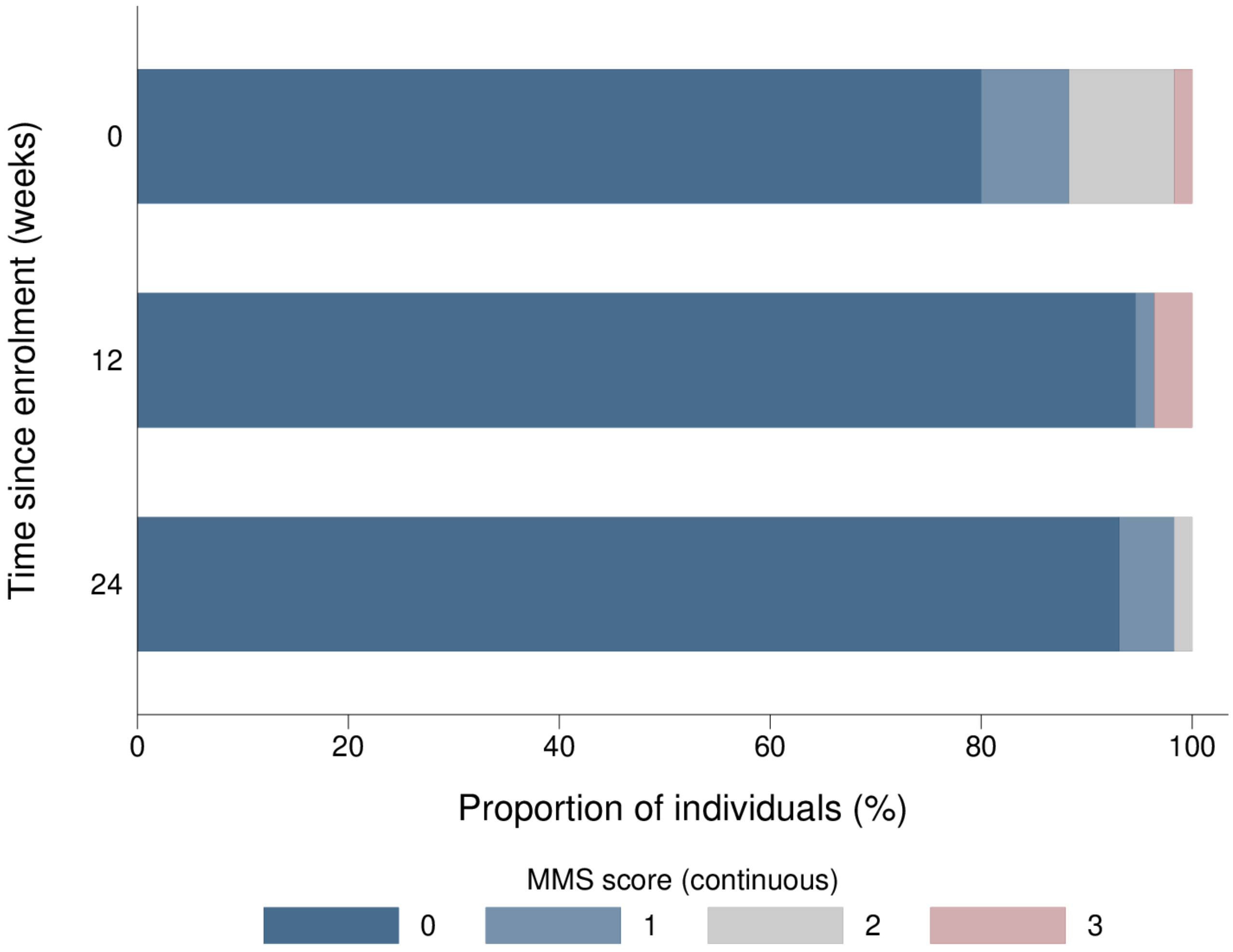

* A score of ≥9 on the MMS has been validated to identify a DSM diagnosis of anxiety, mood or psychotic disorder with 82% specificity and 63% sensitivity[1]

Figure 5. Proportion of individuals with insomnia severity index (ISI) score at each timepoint (continuous measure ranging from 0 to 28*)

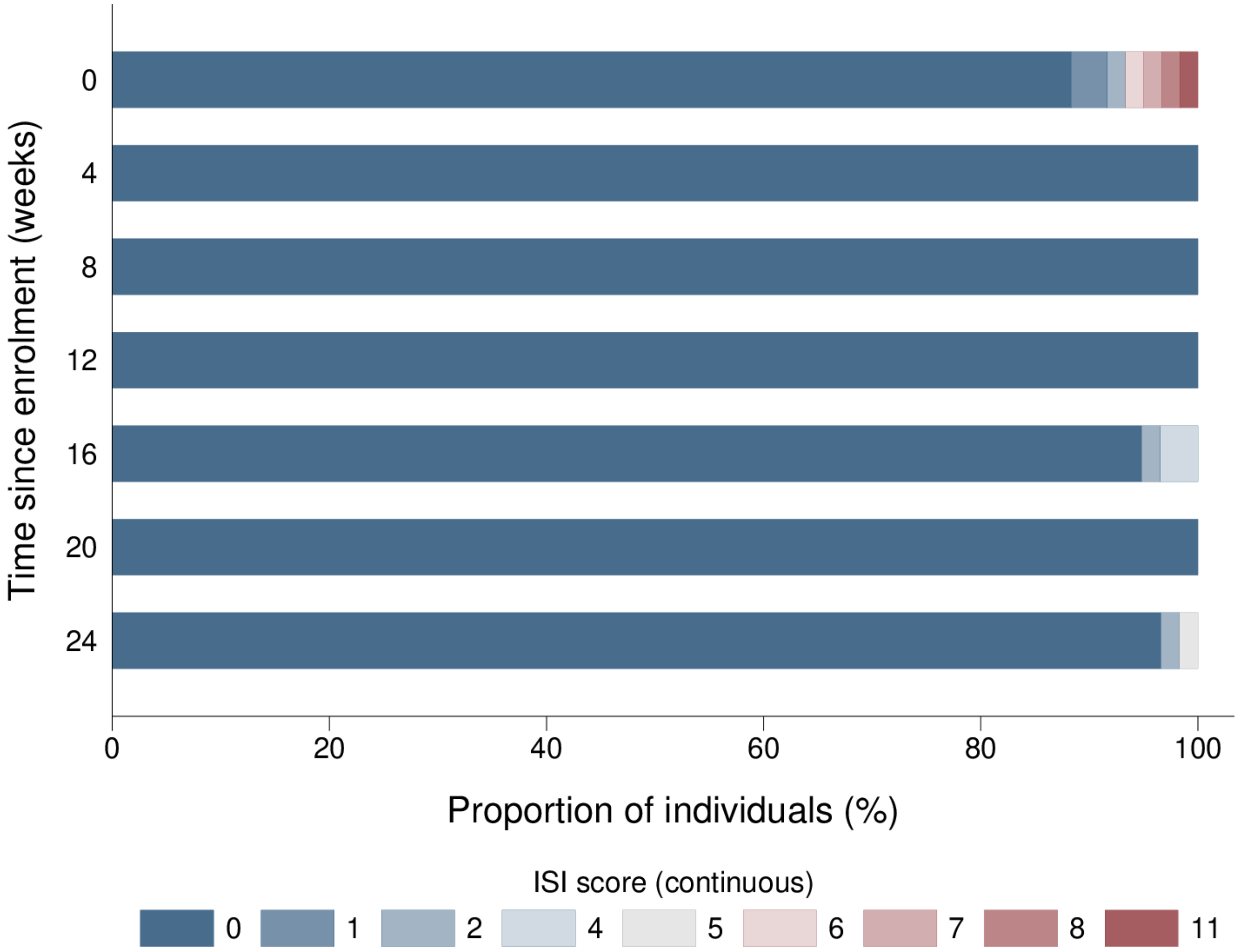

*A score of ≥11 on the ISI has been shown to detect severe insomnia with 97% sensitivity and 100% specificity[2].

Table 9.4 Concomitant medications with potential for dolutegravir interaction taken at any point from baseline to week 24

Concomitant medications	n=60
**Key concomitant preventative medications, n(%)**	
Isoniazid*	40 (67%)
Cotrimoxazole	21 (35%)
**Other concomitant medications with potential for dolutegravir interaction, n(%)**	
Ferrous sulphate	5 (2%)
Metformin	1 (0%)
Rifampicin	1 (0%)
Zinc Sulfate Monohydrate	1 (0%)

*Participants received isoniazid as tuberculosis preventative therapy

References

1. Alexander MJ, Haugland G, Lin SP, Bertollo DN, McCorry FA. Mental health screening in addiction, corrections and social service settings: Validating the MMS. *Int J Ment Health Addict*. 2008;6(1):105-119. doi:10.1007/s11469-007-9100-x

2. Morin CM, Belleville G, Bélanger L, Ivers H. The insomnia severity index: Psychometric indicators to detect insomnia cases and evaluate treatment response. *Sleep*. 2011;34(5):601-608. doi:10.1093/sleep/34.5.601

3. Stanford University. DRM penalty scores and resistance interpretation. HIV Drug Resistance Database. Published 2020. Accessed September 4, 2020. https://hivdb.stanford.edu/page/release-notes/

4. Anderson PL, Liu AY, Castillo-mancilla JR, et al. Intracellular Tenofovir-Diphosphate and Emtricitabine-Triphosphate in Dried Blood Spots following Directly Observed Therapy. *Antimicrob Agents Chemother*. 2018;62(1):1-13.
